# A Novel Intrauterine Device for the Spatio-Temporal Release of Norethindrone Acetate as a Counter-Estrogenic Intervention in the Genitourinary Syndrome of Menopause

**DOI:** 10.3390/pharmaceutics16050587

**Published:** 2024-04-26

**Authors:** Ahmed Abdelgader, Mershen Govender, Pradeep Kumar, Yahya E. Choonara

**Affiliations:** Wits Advanced Drug Delivery Platform Research Unit, Department of Pharmacy and Pharmacology, School of Therapeutic Sciences, Faculty of Health Sciences, University of the Witwatersrand, Johannesburg, 7 York Road, Parktown 2193, South Africa

**Keywords:** implantable devices, intrauterine delivery, controlled release, polycaprolactone, ethyl cellulose

## Abstract

The genitourinary syndrome of menopause (GSM) is a widely occurring condition affecting millions of women worldwide. The current treatment of GSM involves the use of orally or vaginally administered estrogens, often with the risk of endometrial hyperplasia. The utilization of progestogens offers a means to counteract the effects of estrogen on the endometrial tissue, decreasing unwanted side effects and improving therapeutic outcomes. In this study, a norethindrone acetate (NETA)-loaded, hollow, cylindrical, and sustained release platform has been designed, fabricated, and optimized for implantation in the uterine cavity as a counter-estrogenic intervention in the treatment of GSM. The developed system, which comprises ethyl cellulose (EC) and polycaprolactone (PCL), has been statistically optimized using a two-factor, two-level factorial design, with the mechanical properties, degradation, swelling, and in vitro drug release of NETA from the device evaluated. The morphological characteristics of the platform were further investigated through scanning electron microscopy in addition to cytocompatibility studies using NIH/3T3 cells. Results from the statistical design highlighted the platform with the highest NETA load and the EC-to-PCL ratio that exhibited favorable release and weight loss profiles. The drug release data for the optimal formulation were best fitted with the Peppas–Sahlin model, implicating both diffusion and polymer relaxation in the release mechanism, with cell viability results noting that the prepared platform demonstrated favorable cytocompatibility. The significant findings of this study firmly establish the developed platform as a promising candidate for the sustained release of NETA within the uterine cavity. This functionality serves as a counter-estrogenic intervention in the treatment of GSM, with the platform holding potential for further advanced biomedical applications.

## 1. Introduction

The genitourinary syndrome of menopause (GSM) is a condition affecting millions of women worldwide and depicts the wide range of changes that occur during menopause, characterized by a decreased level of estrogen [1,2]. This decreased estrogen level, which leads to anatomical changes within the genitourinary system, a decreased blood flow, less elasticity, thinning of the vaginal tissue and an increased vaginal pH, often results in number of symptoms including vaginal dryness, itching or burning, dysuria, recurrent urinary tract infections and dyspareunia [3,4]. Numerous treatment options are available for GSM including non-hormonal and hormone replacement therapies. Hormonal GSM treatment, which often involves the use of estrogen-containing medications for severe and persistent symptoms, however, presents with several side effects including topical irritation at patch sites, while intravaginal products can induce increased vaginal secretion, spotting, and genital pruritus. Systemic products may additionally induce breast tenderness, vaginal bleeding, nausea, and modest weight gain with other adverse effects including headaches, back pain, abdominal discomfort, and vaginal yeast infections. Notably, unopposed estrogen, without progesterone, triggers uterine lining thickening, fostering endometrial hyperplasia, carcinoma, polyps, and fibroids, attributable to an estrogen–progesterone imbalance [2,3,5,6].

The utilization of progestogens, therefore, offers a means to counteract the effects of estrogen on the endometrial tissue during GSM treatment, thereby reducing the risk of endometrial hyperplasia [5,6]. The oral route is the most common method for administering progestogens in postmenopausal hormone therapy. However, oral administration is associated with first-pass hepatic metabolism, with the extent of metabolism varying based on the chemical structure of each progestogen. To circumvent first-pass hepatic metabolism, alternative routes have been explored, including the intra-muscular, vaginal, percutaneous, intranasal, sublingual, and rectal routes [7,8]. The drawback of systemic progesterone administration, however, is its potential to interact with various steroid receptors, leading to stimulation or blockage of estrogen, androgen, glucocorticoid, and mineralocorticoid receptors, in addition to its action on progesterone receptors, while the most common adverse effect of vaginally administered progestogens is irregular vaginal bleeding. Furthermore, continuous estrogen–progestogen therapy typically results in amenorrhea for most women [7]. Moreover, progestogen administration through transdermal delivery using creams or gels may not achieve sufficient endometrial effects due to lower circulating hormone concentrations. It is also noteworthy that systemic progestogen administration can additionally influence lipid profiles in the bloodstream, dependent on the progestogen type and co-administered estrogen dosage.

The utilization of site-specific, sustained release progesterone, delivered through an implantable drug delivery system (IDDS), therefore offers a means to counteract the effects of estrogen on endometrial tissue, decreasing unwanted side effects associated with current treatments and may further be a viable option for addressing dysfunctional uterine bleeding and menopause-related complications [9].

IDDSs have, in recent times, garnered significant research interest, with a predominant focus on the utilization of polymeric biomaterials in their development [10,11]. IDDSs are specifically designed to be placed within the body, providing the continuous release of a targeted drug, at a controlled rate, over an extended duration. The primary advantages of IDDSs over conventional delivery methods, such as oral and parenteral approaches, include an increased effectiveness and reduced side effects associated with the lower drug doses, thereby promoting superior patient compliance, as well as substantial economic savings within healthcare systems [12,13,14,15,16,17].

IDDSs can be classified into two main types: standalone implants employed for therapeutic purposes (e.g., delivering of contraceptives or treating periodontal and ophthalmic diseases) and secondly as part of a functional implant to enhance the success of the overall implantation procedure by preventing complications like rejection, infection, and inflammation (e.g., in the use of drug-eluting stents or orthopedic implants). Additionally, certain IDDSs combine features of both types, as observed in biodegradable stents and scaffolds used in tissue engineering [16]. IDDSs can be further categorized as either biodegradable or non-biodegradable, depending on the type of polymer employed. Non-biodegradable implants are typically crafted using non-biodegradable polymers, necessitating removal from the body upon drug depletion, with drug release mechanisms being easily predictable due to the stable structure of these polymers. Conversely, biodegradable implants, fabricated from polymers that undergo degradation, have their drug release primarily governed by the rate of polymer degradation [10].

Intrauterine devices (IUDs) are implantable platforms used for a variety of applications including for contraception, as well as for the treatment of various ailments within the female genitourinary system [18,19]. The manufacturing methods employed for IUDs have involved extrusion, 3D printing, and injection molding [20]. The selection of suitable polymers for IUDs is, however, limited due to the requirements of non-swellability and non-biodegradability, or alternatively, a very slow degradation rate. Commercially available IUDs thus primarily employ non-degradable polymers, such as polydimethylsiloxane (PDMS). Mirena^®^, a widely used long-lasting contraceptive IUD, exemplifies this category and is composed of a polyethylene backbone, a drug delivery cylinder, and is enveloped by a rate-controlling PDMS membrane [20].

Norethindrone, a synthetic progestin categorized within the 19-nortestosterone derivatives family, is utilized synergistically with oral contraceptive formulations or as a standalone progestin-only pill. It is efficacious in treating various conditions, including premenstrual syndrome, irregular or painful menstruation, abnormal heavy bleeding, menopausal syndrome, and period postponement. Additionally, it has applications in preventing uterine hemorrhage in complex non-surgical or pre-surgical gynecologic cases and for the management of nonresponsive cyclical mastalgia. Oral norethindrone is characterized by a half-life of approximately 10 h and is administered orally once per day [21].

**Figure 1:**
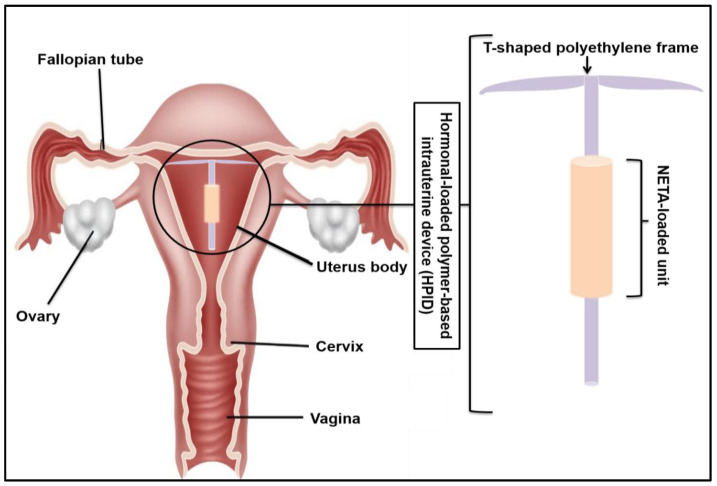
Schematic representation of the polymer-based IUD depicting the NETA-loaded unit placed on an implantable T-shape frame.

This study therefore represents the development, optimization, and evaluation of a polymer-based IUD, loaded with norethindrone acetate (NETA) as a counter-estrogenic intervention in the treatment of GSM. The developed system, which is adaptable to patient-specific estrogen therapy, is prepared from ethyl cellulose (EC) and polycaprolactone (PCL) and has been designed for the sustained release of NETA. Optimization of the system has been achieved using a Design of Experiments (DOE), with extensive in vitro characterization studies performed prior to cytocompatibility studies using the NIH/3T3 cell line. Figure 1 represents the schematic representation of the polymer-based IUD loaded with NETA.

## 2. Materials and Methods

### 2.1. Materials

PCL Mn  =  80,000 was purchased from Sigma-Aldrich (Tokyo, Japan), ethyl cellulose from Sigma-Aldrich Chemie GmbH (St Louis, MI, USA), Norethindrone acetate from Leap Chem (Hong Kong, China) and sodium hydroxide pellets from Merck Chemicals (Modderfontein, South Africa). NIH/3T3 mouse fibroblast cells (ATCC CRL-1658) were obtained from the ATCC (American Type Culture Collection, Manassas, VA, USA), Dulbecco’s Modified Eagle Medium (DMEM) from Life Technologies Limited “Gibco” (Paisley, UK), Fetal bovine serum from PAN Biotech (Aidenbach, Germany), and MTT solution and solubilizing buffer from Roche Diagnostic GmbH (Mannheim, Germany). All other chemicals were of analytical grade and used without further purification.

### 2.2. Methods

#### 2.2.1. Fabrication of the NETA-Loaded Polymeric Matrix

**Figure 2:**
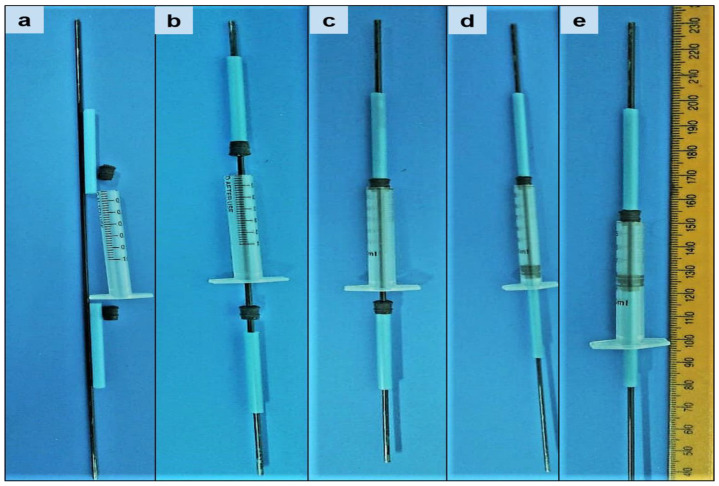
Illustrations of the customized mold (**a**) and technique employed in constructing the NETA-loaded segments (**b**–**e**).

**Figure 3:**
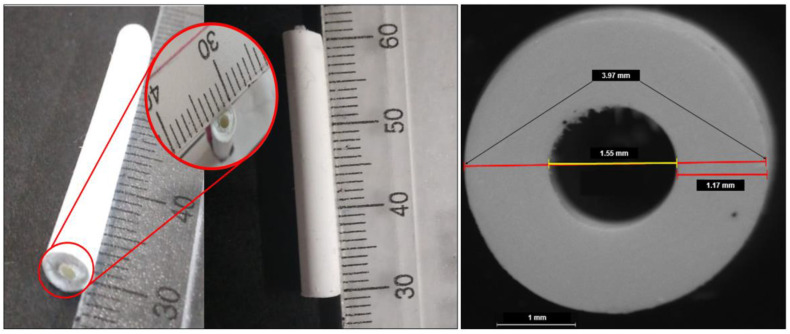
Digital images of the formulated EC/PCL hollow cylindrical segment.

The NETA-loaded polymeric matrix was prepared utilizing the method as described by Pathak et al. [22], with modifications. Briefly, a binary polymer mixture of 30% *w*/*v* concentration was prepared as per the DOE formulations (Table 1) by dissolving different polymers (PCL and EC) ratios in acetone at 45 °C using an oil bath. EC was added first to the acetone prior to the PCL granules 6 min later. NETA (5, 7.5 or 10% *w*/*w*) was added as fine powder to the molten polymers blends of their respective formulations after 25 min and thereafter mixed using a glass rod until a homogenous mixture was achieved. The resultant malleable drug/polymer blends were then fed manually into a customized mold consisting of a 1 mL polypropylene syringe body, equipped with 1 mm central metal rod serving as a centering device (Figure 2). Subsequently, the blends were rapidly cooled at −80 °C to induce polymer crystallization. After 4 h, the hardened segments were removed from the molds and stored at −80 °C overnight. The prepared segments were thereafter dried under ambient conditions in a fume hood for a further 72 h. The prepared dried segments were thereafter visually inspected for any surface defect such as excess material, texture, under-fill, and air bubbles, with any irregularity leading to segment elimination. Thereafter, the regular hollow cylindrical segments were cut using a scalpel into their respective required length and then capped from both ends using 5% PCL solution in acetone (Figure 3). The prepared segments were stored at 20 °C until further analysis.

A 2^2^ factorial design (Design Expert ^®^ DOE software, Version 8, StatEase, Inc, Minneapolis, MN 55413, USA) was employed to investigate and optimize the effect of the independent variables (NETA load (%) and EC-to-PCL (%)) on the critical attributes (percentages of drug released and weight loss) of the NETA-loaded polymeric matrix. The factorial design involved 7 runs, with 4 factorial formulations at low (−1) and high (+1) levels (N1–N4) and 3 central points (N5–N7), as indicated in Table 1. The 3 center point’s replicates were conducted to ascertain the curvature of the data. For all instances, the order of the experiments was fully randomized. Furthermore, for comparative purposes, drug-free formulations corresponding to the design formulations were also prepared.

Formulation variables and processes employed to prepare the NETA-loaded polymeric matrix were selected based on previously reported studies. Various researchers have reported PCL as a base for matrices for insertion via the female genitourinary tract either intravaginally or intrauterine [20,22,23,24,25,26,27,28]. The proportion of polymers used can influence various aspects of the fabrication process, including demolding, which may result in uneven and fractured segment surfaces, potentially leading to sub-optimal release profiles. The range of polymer ratios considered herein were identified based on ratios explored during the preliminary study phase, which yielded smoother segment surfaces, primarily attributed to improved ease of processing. In addition, the melting duration and the selection of a suitable solvent to assist the melting process played a crucial role in determining the feasibility of the early prototypes’ preparation process for this matrix. It was observed that the optimal time required to completely melt the binary polymer blend and drugs at the specified temperature (45 °C) was 30 min. Also, when acetone is utilized as the melting solvent, the blend’s consistency and adaptability for processing are notably improved, in contrast to chloroform or dichloromethane, which resulted in a blend with low consistency, making it challenging to mold and prone to a delayed hardening process during the cooling phase of preparation. Furthermore, the time required to introduce the melted blend into the mold was considered. It was noted that the feeding time into the small-diameter opening mold should be as brief as possible, ideally within a minute, particularly when working with formulations containing a high PCL ratio, as the blend tends to solidify rapidly.

#### 2.2.2. Experimental Design and Optimization

##### Drug Content Uniformity

Prior to the evaluation of each design formulation, the diameter of each hollow cylindrical segment was determined utilizing a digital vernier caliper. For the accurate delivery of dose and to ensure uniform drug distribution within the segment, percent content uniformity was assessed using a slightly modified method as described in the literature [29,30]. Briefly, segment pieces of a 2 mm length were carefully dissected using a scalpel from three regions of randomly selected segments from each formulation. Specifically, the pieces were taken from the middle and from a quarter from each edge. Each piece was thereafter weighed accurately and dissolved in 2 mL acetonitrile in a 20 mL test tube placed in a 40 °C oil bath. Methanol (8 mL) was thereafter added to the mixture to precipitate the polymers. The resultant suspension was then subjected to centrifugation for 10 min at 12,000 rpm (Centrifuge 5810R, Eppendorf, Hamburg-Nord, Germany), with the supernatant filtered through a 0.45 µm PVDF filter and analyzed using an Implen NanoPhotometer^®^ NP80 (Implen GmbH, München, Germany) at 240 nm (ɛ = 0.0471; R^2^ = 0.999). All experiments were conducted in triplicate, with the drug loads determined as the detected mass of drug relative to the total mass of the piece, expressed as mean values with standard deviation (SD).

Validation of the UV-spectroscopy quantification method for specificity to NETA was undertaken utilizing a wavelength scan spanning 200–800 nm conducted on a 40 µg/mL NETA in acetonitrile and methanol (20:80) solution and at the maximum wavelength (λ_max_) of NETA (240 nm). Following this, a solution of a 1:1 ratio of EC to PCL matrix (melted in acetone at the same processing temperature) was prepared at a concentration of 632 µg/mL, centrifuged, and filtered to remove any undissolved polymer, and thereafter analyzed over 200–800 nm and at 240 nm. The higher concentration of EC/PCL was analyzed due to the high polymer to drug ratio utilized in the experimental design. Analysis of the EC/PCL solution wavescan revealed no discernible peak at 240 nm, indicating the absence of interference between NETA and the employed polymers at this wavelength with the absorbance of the polymer solution (0.021) determined to be negligible when compared to that of the NETA solution (2.004).

The validation protocol was repeated using a 40 µg/mL NETA and a 632 µg/mL EC/PCL in simulated uterine fluid (SUF; biodegradation and in vitro drug release media) solution, with no detectable peak similarly determined for EC and PCL at 240 nm. The absorbance of the EC/PCL-SUF solution was further determined to be negligible (0.082) when compared to the NETA-SUF solution (2.030). This analysis therefore determined that the utilized UV-spectroscopy method was specific to NETA and that there was no interference between NETA and the employed polymers at the analysis wavelength of 240 nm, even at higher polymer concentrations. The wavelength scans of the NETA and EC/PCL acetonitrile/methanol and SUF solutions have been provided in the supplementary information (Figures S1–S4).

##### Biodegradation and Accelerated Alkaline Degradation Studies

The biodegradation of the NETA-loaded hollow cylindrical segments in SUF was determined by placing a 5 mm length of accurately weighed segment ($W_{i}$) in 15 mL SUF (pH 7.0; 37 °C) rotating in an orbital shaker at 25 rpm for 8 weeks. SUF (pH 7.0) was prepared based on the method as described by Jinying et al. [31]. The degradation medium was changed and replaced with fresh SUF every 7 days. At a predefined time (8 weeks), the hollow cylindrical segments were removed from the degradation medium, rinsed with double-distilled water to eliminate any residual solutes adhering to the segment surface and allowed to air-dry for 72 h in a fume hood before being weighed ($W_{F}$).

Similarly, the in vitro hydrolysis degradation patterns of NETA-loaded and drug-free hollow cylindrical segments were studied at accelerated alkaline conditions by adapting the method previously developed by Boia et al. [32]. For this study, three samples of 2 mm length for each iteration were weighed accurately and kept immersed overnight in bi-distilled water. Subsequently, the samples were immersed in 3 mL of NaOH solution (5 M) for 10 min at room temperature, gently wiped with filter paper and weighed to obtain the initial weight ($W_{i}$). The samples were thereafter placed in an orbital shaker (37 °C; 25 rpm), removed at predefined time intervals (4, 24, 48, 72, and 96 h), wiped with filter paper to remove excess media, and thereafter weighed ($W_{F}$) at each time point. Biodegradation and accelerated alkaline degradation were thereafter calculated in terms of percentage weight loss using Equation (1):

(1) $$Weight\;loss\;\%=\frac{\left(W_{i}-W_{F}\right)}{W_{i}}\times 100$$

where *W_i_* is the dry initial weight of the segment, and *W_F_* is the dry weight of the segment after “t” incubation in SUF or NaOH. All the experiments were performed in triplicate with all values expressed as the mean ± SD.

##### Textural Analysis

Textural profiling was employed to investigate the hardness of the design formulations (n = 3). To achieve this, a calibrated textural analyzer (TA.XT.plus; Stable Micro Systems, Surrey, UK) equipped with a 5 mm diameter ball probe indenter was employed to press the surface of horizontally mounted hollow cylindrical segment with an average 4 mm diameter, pre-cut to a length of 5 mm. Data acquisition was accomplished using the Texture Exponent Software (Version 6.1.16.0). Moreover, the maximum force generated during the indentation of the segment matrix at a depth of 0.3 mm was determined and regarded as the segment’s hardness or indentation resistance. As a comparison, the hardness of the corresponding drug-free formulation was determined.

##### In Vitro Drug Release Analysis

In vitro drug release studies were carried out on the design formulations (n = 3) at 37 °C in SUF over a 4-week period. For the analysis, each segment was enveloped on a 3D printed T-shaped plastic frame. All in vitro release analyses were carried in 15 mL SUF, placed in an orbital shaker rotating at 25 rpm to facilitate release conditions. At 24 h periodic intervals, 12 mL of the release medium (representing 80% of total release medium) was withdrawn and replenished with an identical volume of fresh medium to maintain sink conditions. All extracted samples were thereafter filtered analyzed by UV spectroscopy at 240 nm (ε = 0.01; R^2^ = 0.997).

##### Statistical Optimization

Statistic optimization of the NETA-loaded segments was achieved utilizing the data generated in the evaluation of design formulations. The parameters utilized for the optimization were the “in range” for the NETA load and EC-to-PCL ratio. In addition, the minimized release and weight loss were set as desirable. Statistic optimization through the DOE generated an optimal formulation of high NETA load (10%) and EC-to-PCL ratio (50%). The optimal formulation was thereafter subjected to content uniformity studies, textural profiling in the wet state (incubated in SUF at pH 7.0, 37 °C for 24 h), and in vitro drug release analysis, as described previously, with hydration studies, structural profiling, morphological analysis, release kinetic modeling and cytocompatibility studies undertaken as described below.

#### 2.2.3. In Vitro Characterization of the Optimal NETA-Loaded Segment

##### Water Retention Capacity

The hydration properties of both the drug-free and optimal NETA-loaded segments were assessed using a gravimetric method. Initially, the samples were weighed in their dry state on a digital balance (Mettler Toledo, Greifensee, Switzerland). Subsequently, all samples were placed in Petri dishes containing purified water maintained within a constant temperature environment at 22 °C. After incubation, the samples were removed, with the excess water extracted using filter paper. The hydration percentage was thereafter calculated using Equation (2):

(2) $$Hydration\;\%=\frac{W_{F}-W_{i}}{W_{i}}\times 100$$

where *W_F_* represents the weight of the samples after incubation in water, and *W_i_* denotes the weight of the initial dry samples.

##### Structural Profiling

FTIR analysis was performed on the optimal NETA-loaded formulation, a drug-free optimal formulation, pure NETA drug, and the pure polymer components (EC and PCL) using an FTIR spectrophotometer (PerkinElmer spectrum 100 FT-IR Spectrometer, Perkin Elmer, Waltham, MA, USA). All FTIR analyses were undertaken over a range of 4000–650 cm^−1^ at room temperature for 20 scans.

##### Morphological Analysis

The surface morphology and the cross-section of the drug-free and optimal NETA-loaded hollow cylindrical segment before and after an 8-week incubation in SUF was analyzed under a scanning electron microscope (ZEISS SEM, Carl Zeiss Microscopy Ltd., Cambridge, UK). Prior to imaging, the surface and cross-section of each sample were mounted on SEM stubs and sputter-coated with gold–palladium [15,29]. SEM images were thereafter obtained at an accelerated voltage of 3 kV at varying magnifications.

##### Mathematical Modeling of the Release Kinetics

Mathematical modeling of the in vitro release data of the optimized NETA-loaded formulation was undertaken to gain a comprehensive understanding of its drug release mechanism. Consequently, five mathematical kinetic models, implemented with DDSolver^®^ software (Version 9.9), an add-in integrated program for Microsoft Excel^®^ (Microsoft, Santa Rosa, CA, USA), were employed to model the experimental release data. For this analysis, the release data was fitted using the zero-order, first-order, Higuchi, Korsmeyer–Peppas, and Peppas–Sahlin models.

In zero-order kinetics, drug release from a formulation occurs at a constant rate. This is illustrated by plotting the fraction of drug released against time [33]. The mathematical expression for zero-order kinetics is

(3) $$F=k_{0}\times t$$

where $k_{0}$ represents the zero-order release constant [34].

The first-order release kinetics delineate a phenomenon wherein the rate of drug release correlates directly with the drug concentration. This is elucidated by plotting the natural logarithm of the remaining drug percentage against time [33]. The mathematical representation for first-order release kinetics is expressed by

(4) $$F=100\times (1-e^{k_{1}t})$$

wherein $k_{1}$ signifies the first-order release constant [34].

The Higuchi model accounts for both dissolution and diffusion in drug release from a drug delivery system. It involves plotting the fraction of drug release against the square root of time [33]. The Higuchi release equation is expressed as

(5) $$F=k_{H}\times t^{0.5}$$

where $k_{H}$ represents the Higuchi release constant [34].

The Korsmeyer–Peppas model characterizes drug release from polymeric systems, with the release rate being exponentially related to elapsed time [33]. The Korsmeyer–Peppas equation is delineated as

(6) $$F=k_{KP}\times t^{n}$$

where $k_{KP}$ represents the release constant incorporating structural and geometric characteristics of each polymeric segment and drug and n is an exponent kinetic constant that reflects the release mechanism [34].

The Peppas–Sahlin model is derived from the concept that it is feasible to discern the dual contribution release mechanisms, diffusional and relaxational, within an anomalous drug release process. In this model equation, the first term signifies the Fickian diffusional contribution, while the second term denotes the Case II relaxational contribution [35]:

(7) $$F=k_{1}t^{m}+k_{2}t^{2m}$$

where $k_{1}$ represents the constant associated with Fickian kinetics, $k_{2}$ represents the constant associated with Case-II relaxation kinetics, and m denotes the diffusional exponent applicable to systems of any geometrical configuration that exhibit controlled release [34].

In all cases, *F* is the total cumulative percentage of drug released at time t. According to Korsmeyer–Peppas, the exponents for cylindrical matrices are *n* = 0.45 for Case I or Fickian diffusion, *n* = 0.89 for Case II transport and 0.45 < *n* < 0.89 for anomalous behavior or non-Fickian transport [36]. After successful modeling, the coefficient of correlation (R^2^), adjusted coefficient of correlation (adjusted-R^2^), Root-Mean-Squared Error (RMSE), Akaike Information Criterion (AIC), and Model Selection Criterion (MSC) were determined, and the kinetic parameters obtained.

### 2.3. Cytocompatibility Studies

#### 2.3.1. Cell Culture and Experimental Setup

In vitro cytocompatibility studies were undertaken by determining the cell viability of NIH/3T3 mouse fibroblast cells. At its core, the primary objective of this assessment was to discern any potential cellular detriment induced by the prepared NETA-loaded segment. The utilization of a cell line differing from those originating from the uterus, was rooted in the study’s emphasis on elucidating the drug cytotoxicity and its interactions with the polymeric matrix, rather than assessing any uterine-specific effects [37]. Additionally, previous studies for the treatment of endometriosis have utilized NIH/3T3 cells to probe the cytotoxicity of their respective delivery systems [38,39,40], accordingly highlighting the role of NIH/3T3 cells in elucidating the cytotoxicity of various delivery modalities.

For the cytocompatibility study, NIH/3T3 cells (ATCC CRL-1658) were cultured in Dulbecco’s modified Eagle’s medium (DMEM) containing 10% (*v*/*v*) fetal bovine serum (FBS) and 1% (*v*/*v*) penicillin/streptomycin. All cells were thereafter incubated in a humidified incubator (NUAIRE, Radobio, China) at 37 °C with 5% CO_2_, with the cell media changed every 2 days until reaching ≈ 90% confluency.

#### 2.3.2. Device Segment Sterilization and Sample Preparation

Prior to the performance of the cytocompatibility studies, the drug-free and optimal NETA-loaded segment was exposed to source UV light on a continuous path for 5 min (UV-C 254 nm, UV EQUIP, Midrand, South Africa), washed in 70% ethanol for 15 s, dried, and thereafter handled under aseptic conditions in a Class II biological safety cabinet (ESCO, Singapore) [13]. Sample preparation consisted of extraction of leachates from the prepared drug-free and NETA-loaded segments. Briefly, under sterile conditions the devices were incubated in 10 mL sterile SUF (pH 7) at 37 °C and 25 rpm for 5 days. The supernatant was thereafter filtered through sterile 0.45 μm syringe filters under sterile conditions and were then diluted 1:5 with culture media.

#### 2.3.3. MTT Assay

Cytocompatibility of the drug-free and NETA-loaded segments was evaluated (n = 5) using an MTT assay (3-(4,5-dimethylthiazol-2-yl)-2,5-diphenyl-2H-tetrazolium bromide dye). For this assay, a cell suspension with density of 2 × 10^4^ cells/well in 100 µL was seeded on 96 well plates and incubated for 24 h at 37 °C and 95% relative humidity containing 5% CO_2_. Afterward, the growth medium was aspirated and replaced with a growth medium consisting of the extracted leachates, with the cells thereafter grown for an additional 1, 3, 5 and 7 days. Throughout the 7-day testing period, the growth medium containing the extracted leachates was replenished every 2 days. At the end of each designated time point (1, 3, 5, and 7 days), MTT solution (10 µL) was added to each well plate, followed by an addition of 100 µL solubilizing buffer after 4 h, and incubation overnight under the standard culture conditions. The absorbance’s reading of each treatment sample was thereafter taken in triplicate at 570 nm against a 690 nm background using a VICTORTM X3 Multilabel Plate Reader (PerkinElmer, Waltham, MA, USA). In addition to the treatment arms (the NETA-loaded and drug-free segments), NIH/3T3 cells in culture medium and cells in culture medium with DMSO were used as negative and positive controls, respectively.

### 2.4. Statistical Analysis

This study employed Design Expert^®^ 8 for the construction of FD matrix and to investigate the influence of independent variables on the selected response parameters of the fabricated segments. The obtained results were presented as mean values ± SD, calculated utilizing Microsoft Excel 2010 software. Statistical analysis was further conducted using SPSS version 16.0 for Windows. The Shapiro test was additionally used to evaluate the normal data distribution. Two-sample t-tests were employed to determine significance between the two experimental groups and the analysis of variance (ANOVA) was employed, followed by the application of the Tukey HSD and Games-Howell post hoc tests for result comparisons between three and more experimental groups, with all results at a 95% confidence interval.

## 3. Results and Discussion

### 3.1. Evaluation of the Experimental Design

#### 3.1.1. Drug Content Uniformity and Segment Geometry

The assessment of drug content uniformity offers valuable insight regarding drug loads across the various batches and the drug distribution within each segment. Table 2 provides the average diameter and drug content of the drug-loaded segments. The findings exhibited that the drug content in all formulations closely matched the initial feed ratios for the different batches, with a very low SD, suggesting that NETA was uniformly distributed within their respective polymer matrices, and that there was no substantial drug decomposition that took place during the fabrication process. The uniform distribution of the drug within the matrix is likely a consequence of the rapid cooling of the molten blend, facilitating instant crystallization and polymer phase solidification. This process likely hinders the sedimentation of drug particles, ensuring their uniform dispersion throughout the matrix. These observations align with the results reported by Asvadi et al. [24] in a previous study. Also, from the drug content present, it is evident that the loaded drug is affected by the amount of the drug used for segment preparation. Furthermore, it is observable that the hydrophobicity of the drug facilitates the incorporation within the matrix of the corresponding hydrophobic polymer blend. Moreover, the fabricated segments exhibited quite minimal variation in diameter, with values below 4%. This slight variability is likely attributable to variances in the density and viscosity of the malleable blends utilized across the various formulations. Consequently, when considering the diameter and content uniformity results, it can be deduced that the fabrication technology and processing conditions meet the criteria for reproducibility and are deemed suitable and acceptable [41].

#### 3.1.2. Segment Degradation

The structural alterations linked to degradation could potentially impact the drug release patterns of the prepared hollow cylindrical segments [42]. Consequently, it is crucial to track the in vitro erosion by assessing the percentage weight loss as a function of time. PCL, which is contained within the segment, exhibits a total in vivo degradation time frame spanning from 2 to 4 years, which is primarily driven by the random hydrolytic cleavage of ester linkages, leading to a reduction in its molecular weight to below 3000, and subsequently, intracellular degradation takes place [32,43].

**Figure 4:**
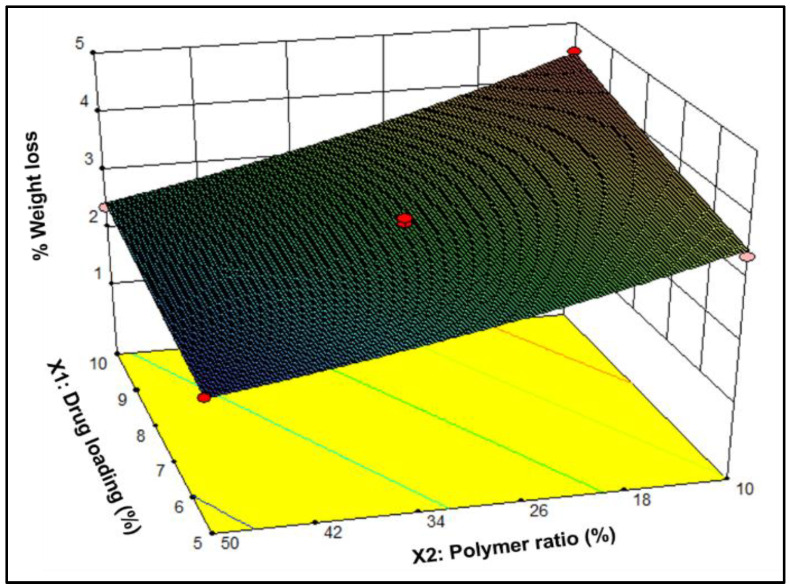
Three-dimensional surface plot depicting the influence of drug load (%) and EC-to-PCL ratio (%) on the weight loss percentage of the NETA-loaded segment.

The findings pertaining to the percentage weight loss throughout the 8 weeks of incubation in SUF of formulations N1 to N7 exhibited a range from 1.92 ± 0.06 to 4.51 ± 0.21%, as depicted in Figure 4. Notably, at 50% EC-to-PCL, as exemplified by formulations N3 and N4, it was noted that this polymer ratio was associated with a significant increase in weight loss with the transitioning of the drug load from 5% (N3) to 10% (N4). In addition, maintaining the drug load at a higher level (10%) with a decrease in the EC level led to a significant increase in weight loss as evidenced when comparing formulation N4 and N2. Furthermore, at 10% EC-to-PCL levels, altering the drug load from 5% to 10% led to an increased weight loss, as observed when comparing N1 and N2 formulations. Moreover, it is worth noting that the combinations of intermediate levels for both independent variables resulted in an intermediate weight loss.

**Figure 5:**
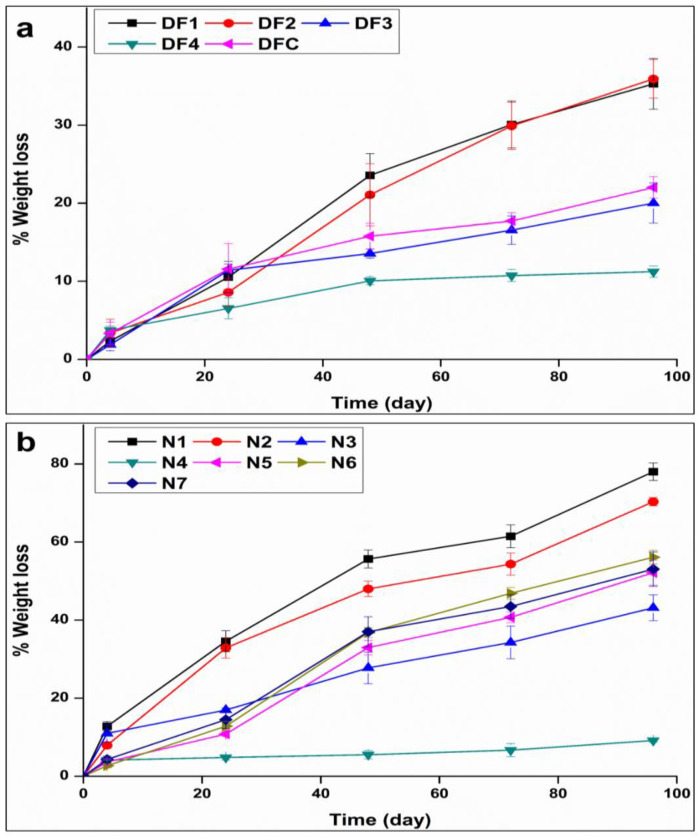
Accelerated weight loss (%) in alkaline media as a function of time: (**a**) Drug-free segment and (**b**) NETA-loaded segment. DFC represents the drug free formulation with a 30% EC to PCL ratio.

Alkaline degradation studies were additionally conducted on both drug-free and optimal NETA-loaded hollow cylindrical segments under high pH conditions (5 M NaOH), with the influence of EC-to-PCL ratios and drug loads on the degradation rates examined and compared. Figure 5 illustrates the results in terms of percentage weight loss. Notably, formulations drug-free 1 (DF1) and N1 exhibited the highest degree of degradation, while formulations DF4 and N4 displayed the least degradation over the same duration. These results highlighted a decrease in the degree of weight loss with an increase in the EC-to-PCL percentage. Regarding drug load, a distinct trend emerged when comparing the degradation of DF formulations with low EC-to-PCL ratios (DF1 and DF2) after 96 h to their corresponding formulations loaded with drug. The NETA-loaded formulations (N1 and N2) demonstrated a significant increase in weight loss% when compared to the drug-free counterparts (*p* < 0.05). Conversely, when examining the DF formulation with high EC content (DF4) in comparison to its counterpart with both high EC content and high drug load (N4), the findings reveal no significant differences in weight loss percentage after 96 h (*p* > 0.05).

From these results, it can be inferred that the compositions used in the preparation of the segments exert a significant influence on the weight loss profile and erosion characteristics. EC, characterized by its hydrophobic nature, high molecular weight, and chain stiffness, plays a crucial role in these observations. As the content of ethyl cellulose increases within the matrix, there is a concurrent rise in hydrophobicity. This heightened hydrophobicity establishes a protective barrier that restricts the penetration of water or other degrading agents into the matrix, consequently decelerating the degradation process. Moreover, the augmentation of EC content can enhance the interfacial adhesion between EC and PCL. This improved adhesion fosters a more compact and uniform structural arrangement, resulting in fewer micro-voids or weak points within the segments. As a result, it reduces the availability of sites for water ingress and, consequently, hinders the initiation and progression of the degradation processes. The combined effects of heightened hydrophobicity and improved interfacial adhesion contribute to the observed reduction in weight loss and erosion characteristics in the segments.

#### 3.1.3. Textural Profiling

In the context of long-term loading, it is imperative for implants to exhibit robust mechanical properties, enabling them to effectively withstand persistent stress and mitigate the risk of undesirable deformations [16]. In this regard, the hollow cylindrical segments, tailored for intrauterine deployment, necessitate adequate strength and flexibility to endure internal uterine environment without detrimental effects on surrounding tissues, as well as to expedited removal during emergency scenarios. Consequently, an assessment of the mechanical properties of the fabricated segments in dry state as well as for the optimal formulation in wet state was conducted by quantifying the externally applied stress required to induce deformation in the prepared segments.

**Figure 6:**
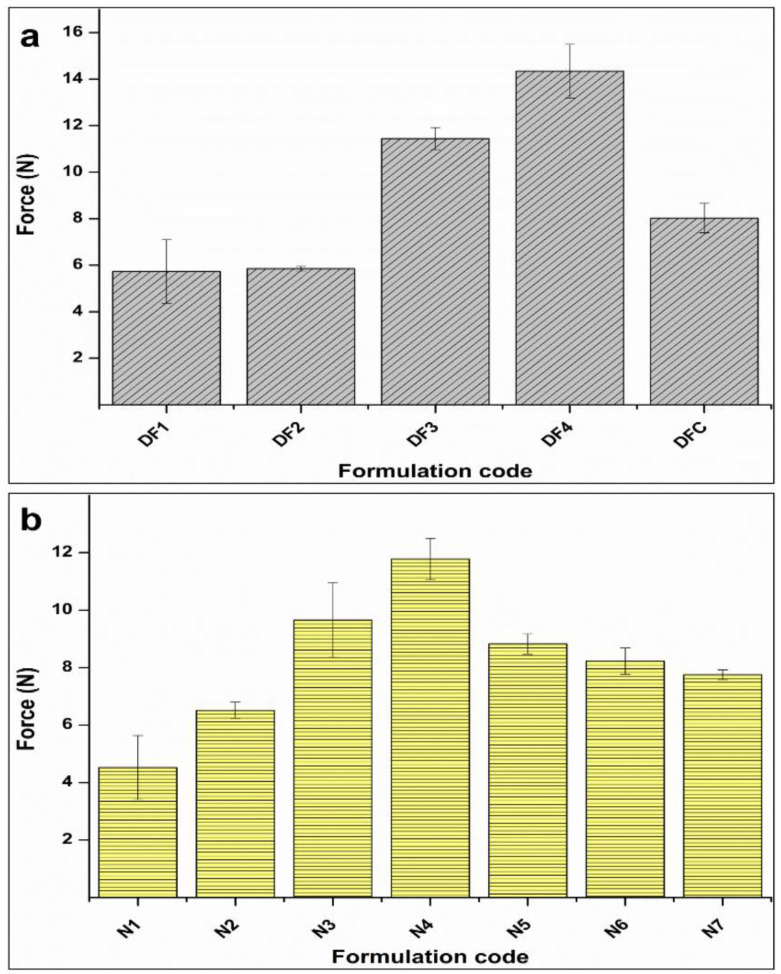
Analysis of indentation force of the drug-free segment (**a**) and NETA-loaded segments (**b**).

Figure 6 illustrates the force values (N) generated from the penetration of the probe into the various segment matrices. The findings from the drug-free segment reveal a statistically significant impact of the increasing level of EC on the force (Figure 6a). This effect becomes evident when contrasting formulations DF4 with the formulations featuring 30% and 10% of EC-to-PCL (*p* < 0.05). Furthermore, formulation DFC, characterized by a medium level of EC-to-PCL (30%), demonstrated a slight, albeit non-significant, increase in force compared to DF1 and DF2 (low level of EC) (*p* > 0.05).

In the context of NETA-loaded segment (Figure 6b), the findings indicate a noteworthy influence of escalating EC content on the indentation force, as evidenced by a statistically significant increase observed in formulations N4 and N3 in comparison to N1, as well as when contrasting N4 with other formulations (*p* < 0.05). An elevation in drug load further demonstrated a slight, non-significant, increase in force, exemplified in the comparison between N1 and N2, as well as in formulations at center points, and the comparison between N3 and N4 (*p* > 0.05).

The hydrated optimal NETA-loaded formulation and the corresponding drug free formulation, after 24 h incubation in the SUF, exhibited resistance to indentation of 14.3 ± 0.8 and 11.44 ± 1.4 N. These results demonstrate statistically significant increase in hardness due to the hydration of the drug free formulation (*p* = 0.041), while there is no difference between the dry and hydrated NETA-loaded formulations (*p* = 0.078), with only a slight increase in the hardness seen. The observed heightened resistance to indentation, as deduced from the preceding results, may be attributed to the uniform dispersion of drug and EC within the PCL matrix. This uniform dispersion likely contributes to improved interfacial adhesion between the polymers and drugs, specifically pertaining to their hydrophobic nature, thereby resulting in a reduction in micro-voids in the interphase region.

#### 3.1.4. In Vitro Drug Release

In vitro measurements necessitate the replication of an environment mirroring human uterine conditions. The intricate composition of human uterine fluid, encompassing inorganic compounds, carbohydrates, lipid factors, amino acids, peptides, and proteins, displays dynamic variations across different menstrual cycle phases and among individuals [44,45]. These intricacies, arise from its metabolic origin, pose formidable challenges for precise simulation. Several endeavors have been undertaken to develop an experimental system capable of simulating this complex milieu such as employing synthetic media as Ringer solution, Hanks medium, and amino acid solutions [44]. In alignment with the current study’s objectives, the SUF was formulated by drawing insights from the pertinent literature [31]. This fluid was tailored to encompass compounds predominantly found in human uterine fluid, offering a methodologically robust approach to emulate the complex uterine environment [44]. Additionally, the pH level in uterine fluid exhibits high variability with reported pH values ranging from ∼6 to 8 [44,46]. In the study by Jinying et al., the release behavior of cupric ion and indomethacin from Cu-IUDs was investigated in SUF with a pH value adjusted around 6.8–7.4 [31]. The postmenopausal period is characterized by microbiota imbalance [6], potentially leading to an elevated pH in the upper genital tract [47]. In our study, a constant pH value of 7 was maintained throughout the experimentation. This standardization was implemented to align with our primary objective, focusing on determining the impact of the EC-to-PCL ratio and drug load on the release weight loss profiles of the prepared platform. Furthermore, the volume of uterine fluid exhibits inter-individual variation and undergoes fluctuations across different menstrual phases, with the reported volumes of 83 µL–180 µL, 105 ± 92 µL, 40 ± 32 µL, and less than 5–35 µL for mid-cycle, follicular phase, luteal phase, and mid-luteal phase, respectively [48]. Herein, we standardized the volume to the lowest value to maintain sink conditions consistently throughout the assessments of drug release and platform weight loss. Moreover, the dynamics of fluid movement within the uterus are predominantly governed by myometrial contractions. However, quantifying the uterine movement and establishing a correlation with the rotational speed in the release assessment remains a methodological challenge. To ensure a reproducible laminar flow and prevent turbulence, agitation should be maintained at a relatively low rate [48]. The choice of 25 rpm was therefore grounded in the need for consistency in our experimental conditions.

**Figure 7:**
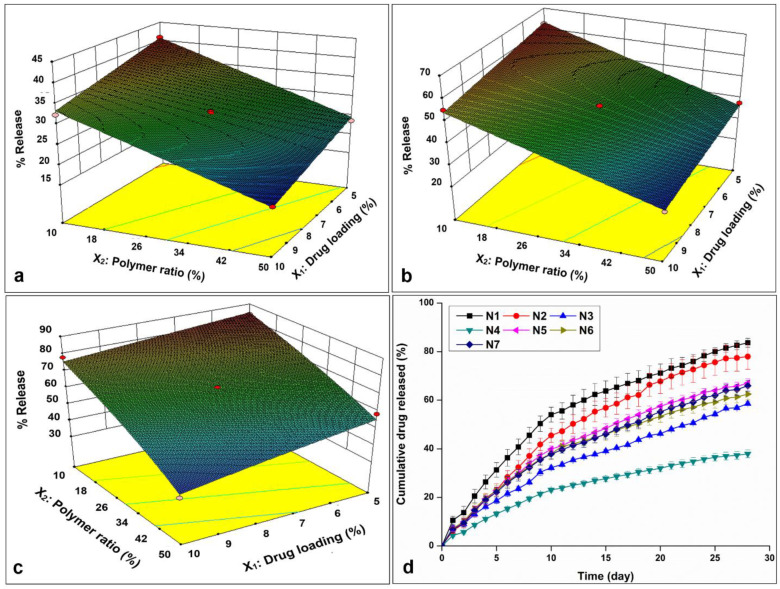
Three-dimensional surface plots depicting the influence of drug load (%) and polymer ratio (%) on the cumulative drug release at (**a**) week 1, (**b**) week 2, and (**c**) week 4, with (**d**) displaying the release profiles of the prepared design formulations.

The cumulative NETA release percentages of the prepared formulations (N1 to N7) over a 4-week period (Y4) exhibited a considerable range, spanning from 37.95 ± 1.62 to 83.67 ± 1.84. Notably, formulations N4, characterized by high drug load (10%) in combination with high EC-to-PCL ratio (50%), exhibited comparatively low release percentages. In contrast, formulation N1, featuring the lowest level of drug load and EC percentage (5% and 10%, respectively), displayed the highest release. Additionally, when examining formulations with the drug load levels at 5%, a distinct pattern emerged as the percentage of drug release increased from 58.6 ± 0.7 (N3) to 83.67 ± 1.84 (N1) with a corresponding transition in the EC percentage from 50% to 10%. Furthermore, formulations distinguished by medium levels of both independent variables (7.5% and 30%, for drug load and EC ratio, respectively) displayed intermediate release percentages, as evidenced in formulations N5, N6, and N7. These findings underscore the profound impact of variations in both independent variables on the percentage of drug release, elucidated in Figure 7a–c, which portrays the main effects of drug load and EC:PCL ratio on the cumulative NETA release percentage over a 4-week evaluation period. The drug release profiles of NETA (Figure 7d) revealed sustained release characteristics from the polymer blend-based segments. Over the 4-week observation period, it is evident that the N1 formulation achieved the highest release, followed by N2, whereas N4 exhibited the lowest release percentage. These results are consistent with observations from a study by Desai et al. [49], which demonstrated that an increased amount of EC led to a reduction in drug release. This reduction was attributed to the increased hydrophobicity of the prepared granules with higher amounts of EC, which impeded the penetration of the release media into the matrix and decreased the diffusion of the drug into the release media. Moreover, these trends were observed in a study investigating progesterone release from PCL matrices [50], where despite a similar release pattern across varied drug loads, formulations with higher drug concentrations exhibited elevated release quantities, while those with lower drug loads revealed higher cumulative drug release percentages.

#### 3.1.5. Data Analysis and Optimization of the Statistical Design

Applying the principles of experimental design, a 2^2^ FD was employed to generate seven formulations of NETA-loaded tubular segments. An overview of the design matrix, which encompasses two independent factors, and the corresponding responses is presented in Table 3. The experimental data pertaining to percentages release after 1, 2, 3 and 4 weeks and weight loss percentage after 8 weeks (denoted as Y1, Y2, Y3, Y4, and Y5, respectively) underwent mathematical transformations, involving the power transformation for Y3 and Y4, as well as the natural logarithm transformation for Y5. Linear regression models were then established and assessed based on the ANOVA results. Notably, the low probability values (*p* < 0.0001, <0.0001, =0.001, =0.0009, and =0.0001, for Y1, Y2, Y3, Y4 and Y5, respectively) highlight the robust statistical significance of the constructed models for responses, verified with a 95% confidence interval. The lack of significant lack-of-fit, as indicated by Lack of Fit *p*-values of 0.0764, 0.4647, 0.2569, 0.3269, and 0.3004 for Y1, Y2, Y3, Y4, and Y5, respectively, further underscores the models’ high predictive capability, suggesting that the lack-of-fit has minimal influence in comparison to random error. The results reveal that the variation of EC-to-PCL ratio (X_2_) exert a significant influence on both drug release throughout the 4 weeks and weight loss (Y5) percentages. Likewise, the drug load variation (X_1_) has been determined to have a statistically significant impact on weight loss and drug release (%), albeit without exerting a statistically significant influence on drug release during week 3, as well as less influences on the responses compared to the EC-to-PCL ratio.

In Table 4, a comprehensive set of fitness statistics is presented. The similarity between the anticipated and observed responses is manifested in the high values of R^2^, denoting more than 0.96. Furthermore, all responses evidenced remarkable levels of precision, surpassing a threshold of 4, and the predicted R^2^ values closely mirrored the adjusted R^2^ values. Additionally, model selection considerations were directed towards the minimization of Prediction Error Sum of Squares (PRESS) values, reinforcing the efficacy of the selected models. Moreover, the analysis of NETA-factorial design results led to the derivation of the following first-order linear equations, expressed in terms of coded factors:

(8) $$Y1=+28.95-3.68{\;X}_{1}-8.08\;{\;X}_{2}$$

(9) $$Y2=+45.47-4.48{\;X}_{1}-13.24\;{\;X}_{2}$$

(10) $${\left(Y3\right)}^{3}=+2.05\times 10^{5}-31487.37{\;X}_{1}-1.462\times 10^{5}{\;X}_{2}$$

(11) $${\left(Y4\right)}^{2}=+4386.46-727.93{\;X}_{1}-2051.98\;{\;X}_{2}$$

(12) $$lnY5\;=+1.07+0.13{\;X}_{1}-0.30\;{\;X}_{2}$$

In Equations (8) and (9), it is evident that both the main effect terms *X*_1_ and *X*_2_ exhibit statistically significant negative influences on the obtained release responses during week 1 and 2 as well as negative effect of *X*_2_ on the cube of the release responses during week 3 as demonstrated in Equation (10). In addition, both main effect terms display significant negative effects on the squired of release responses during week 4 (Equation (11)). Furthermore, as indicated in Equation (12), the main effect term *X*_1_ displays a significant positive effect on the natural logarithm of weight loss percentage, whereas the term *X*_2_ demonstrates a statistically significant negative association with the natural logarithm of weight loss percentage.

### 3.2. Characterization of the Optimized NETA-Loaded Segment

#### 3.2.1. Water Retention Capacity

The in vitro hydration studies of the optimized drug-free and NETA-loaded formulations in water were conducted over 24 h. The drug-free formulation displayed a significantly higher hydration percentage compared to the NETA-loaded formulation (34.63% ± 2.2 vs. 17.52% ± 0.32, respectively; *p* < 0.05). This observation could be attributed to the increased hydrophobicity, reduced porosity [51], and hindered water diffusion resulting from drug depositions on micro-voids and the surface of the NETA-loaded segment.

#### 3.2.2. Structural Profiling

**Figure 8:**
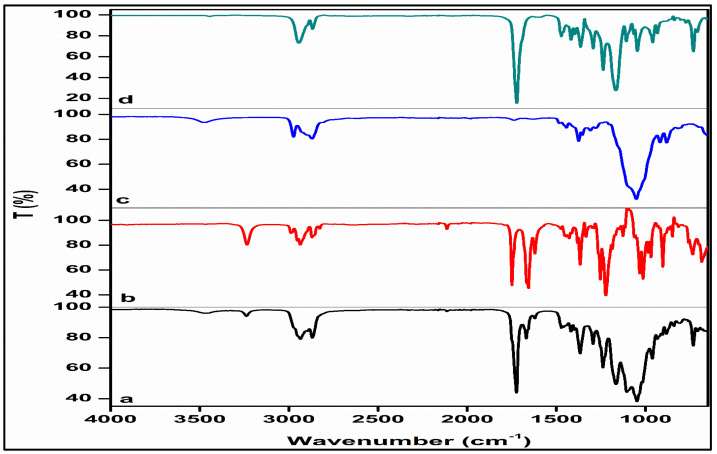
FTIR spectra of (**a**) the optimized NETA-loaded segment, (**b**) NETA, (**c**) EC, and (**d**) PCL.

Evaluation of the FTIR spectrum of PCL revealed key absorption bands at 2943 cm^−1^ and 2867 cm^−1^ related to asymmetric and symmetric stretching vibrations of CH_2_ groups, respectively, and 1721 cm^−1^ attributed to the stretching vibrations of the ester carbonyl group (C=O) (Figure 8). Additionally, the signal located at 1294 cm^−1^ is corresponding to C-O and C-C stretching vibrations in the crystalline phase. Furthermore, distinct signals at 1240 cm^−1^ and 1165 cm^−1^ were detected, associated with the asymmetric and symmetric stretching of C-O-C bonds, respectively [52,53]. The EC spectrum revealed a prominent absorption band at 1054 cm^−1^, and a peak at 1376 cm^−1^ attributed to CH_2_ bending vibrations. The presence of OH groups was confirmed by a less pronounced but discernible peak at 3486 cm^−1^. Furthermore, the spectrum exhibited small yet sharp peaks at 2977 cm^−1^ and 2872 cm^−1^, corresponding to CHO stretching vibrations, which in alignment with results reported previously in the literature [54,55]. The FTIR spectrum of pristine NETA exhibited two distinct peaks. The initial peak at 3235 cm^−1^ corresponds to the O–H stretching of the unbound hydroxyl group, while the subsequent peak at 1650 cm^−1^ corresponds to the C=O stretching of the ketone. These findings align with previous reports in the literature [56]. The NETA-loaded segment’s spectrum revealed the primary bands of NETA, albeit with lower intensities, and bands related to the PCL and EC structures were detected, suggesting no significant changes or shifts in major characteristic peaks. Identical characteristic peaks and the absence of discernible new peaks in the prepared segment compared to the pure drug and polymers indicated homogeneous mixing of the drug and polymers, affirming physical interaction and further supporting the absence of strong chemical interaction. The FTIR spectra data validate the compatibility of segment-forming polymers with the drug, indicating that performance characteristics were not altered.

#### 3.2.3. Morphological Analysis

**Figure 9:**
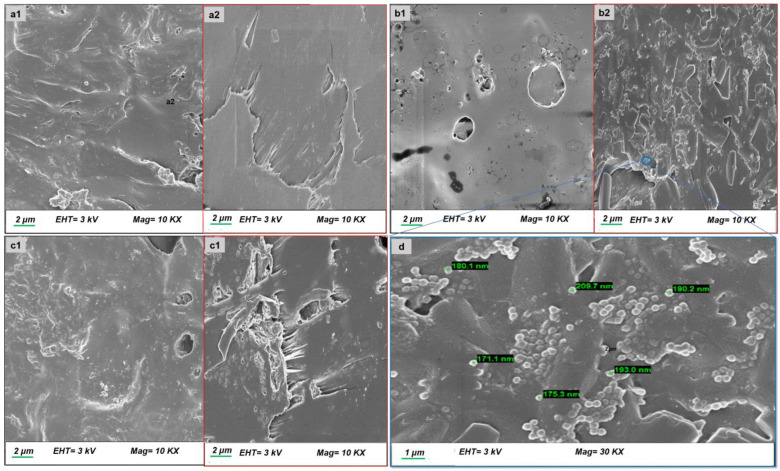
SEM analysis of surface and cross-sectional views for (**a1**,**a2**) the drug-free PCL-EC-based segment; (**b1**,**b2**) the NETA-loaded segment; (**c1**,**c2**) 2-month degraded NETA-loaded segment; and (**d**) precipitated NETA-loaded nanoparticles in the cross-section of the NETA-loaded segment.

Assessments of morphological features were conducted using SEM to investigate the surface structural changes in the optimized drug-free and drug-loaded segments, as well as the alterations observed due to the erosion during an 8-week incubation in SUF (Figure 9). SEM analysis revealed that prior to drug loading, the drug-free EC/PCL segment exhibited a somewhat roughened surface and cross-section with a sea-wave-like structure, characterized by fewer cavities, voids, and pores (Figure 9(a1,a2)). These surface irregularities and cavities lessened in the NETA-loaded segments without any apparent signs of drug migration on the segment surface (Figure 9(b1,b2)). Following an 8-week degradation period in SUF, the NETA-loaded segment exhibited cracked and rough surfaces (Figure 9(c1,c2)). Furthermore, the cross-section contained closed-type pores that were not interconnected and lacked channel-like structures, which may limit the diffusion of the dissolution medium into the segment matrix. In an interesting observation, nano-crystalline structure, measuring in the range of 170 to 210 nm, appeared uniformly distributed within the cavities of the NETA-loaded segment’s cross-section (Figure 9d). This observation can be attributed to the rapid cooling of the NETA-loaded segment from the molten state to −80 °C during the fabrication process, which prevented the growth of the grains noted in the other segments, promoting the formation of these nano-structures [57].

#### 3.2.4. Drug Release and Release Kinetics of Optimized Formulation

The efficacy of a pharmaceutical dosage form crucially relies on the processes of drug release and dissolution within biological fluids. The dissolution of a drug is a complex interplay of factors, including drug and excipient wettability and solubility. The predominant mechanisms governing drug release from polymeric matrices typically comprise diffusion, erosion, and swelling, with the prevalence of a particular mechanism contingent upon the nature of the polymer employed [58,59]. Hydrophilic polymer-based matrices enable liberation of the drug load through the ingress of water into the system [60], a process often sensitive to temperature variations and transitions from a glassy to a matrix relaxation state. Consequently, in such matrices, the phenomena of swelling and diffusion frequently manifest. Conversely, in the case of hydrophobic polymer-based matrices, drug release predominantly occurs via diffusion or erosion, with one mechanism prevailing over the other based on the properties of the drug and the utilized excipients [61].

**Figure 10:**
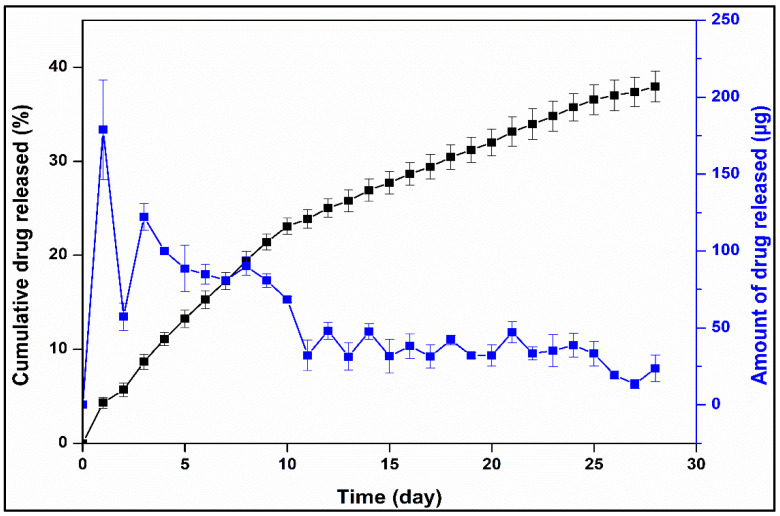
NETA release profile from the optimal formulation.

The drug release profiles of NETA revealed sustained release characteristics from the polymer blend-based segments with the optimal formulation displaying a favorable drug release profile, featuring a relatively low cumulative release percentage of approximately 38% over the 4-week testing period. However, it is noteworthy that there is an initial burst release on day 1, amounting to 178 μg, followed by a gradual decrease in daily drug release up to day 10 and thereafter extended release up to day 28 (Figure 10). The occurrence of burst release can be attributed to various chemical, physical, and processing factors. Factors such as drug migration during the cooling and drying processes resulted in non-uniform drug distribution, thereby promoting burst release. Additionally, the development of surface cracks is another factor contributing to burst release, as it facilitates surface erosion [62]. Kumar et al. investigated the NETA release rate, suitable for intrauterine administration, from a cylindrical-shaped pellet (1.47 mm diameter and 4 mm length) or a crystalline NETA powder placed inside 5 mm length and 3.18 mm diameter silastic capsule. The findings showed in vitro release rate comparable to that of optimal NETA-loaded matrix, as the crystalline system and the cylindrical-shaped pellet exhibited an initial release rate of 43 µg/day and 25 µg/day which decreased to 23.7 µg/day 16.8 µg/day on day 12, respectively [63].

The pharmaceutical field places significant emphasis on the quantitative assessment of drug release from delivery systems. To gain a deeper understanding of this process, various mathematical models have been developed to fit experimental data. These models are designed to depict release profiles by comparing released drug fractions over time [61]. To elucidate the drug release mechanism from the developed NETA-loaded segment, the release kinetics of the optimized formulation were assessed through fitting the data to several release kinetic models. Table 5 presents the goodness of fit outcomes, including R^2^, adjusted R^2^, RMSE, AIC, and MSC values, applied to the averaged release profiles.

Among the mathematical models applied to describe the drug release behavior of the optimal formulation, the Peppas-Sahlin model exhibited the best fit, followed by the Korsmeyer–Peppas model. Peppas-Sahlin model demonstrates a satisfactory fit, owing to its ability to integrate both diffusional behavior and case II relaxation behavior. When the relaxation mechanism is negligible, the equality of the “n” value in the Korsmeyer-Peppas model and the “m” value in the Peppas-Sahlin model is expected [62]. Hence, the disparity between n (0.592) and “m” (0.801) exponents underscores that the mechanism governing NETA release is contingent on both diffusion and case II relaxation. Moreover, the obtained release exponent n from the Korsmeyer–Peppas model was 0.592 further confirms that the mechanism of NETA release exhibits an anomalous behavior or non-Fickian transport, implying that the release kinetics are characterized by a degree of complexity with several internal collective processes possibly interplay beside the diffusional mechanism, including polymer erosion, relaxation and swelling. In addition, the high value of Korsmeyer–Peppas kKP parameter (5.465) suggests that a burst release occurred during the initial phase of the release (approximately 178 µg of NETA being released on Day 1).

### 3.3. Cytocompatibility Studies

The in vitro cytocompatibility of the optimized NETA-loaded segment and its corresponding drug-free counterpart was assessed by determining the cell viability of the NIH/3T3 cells. The cell culture medium served as a negative control, with resulting cell viability set at a value of 100% for comparison with the test samples.

**Figure 11:**
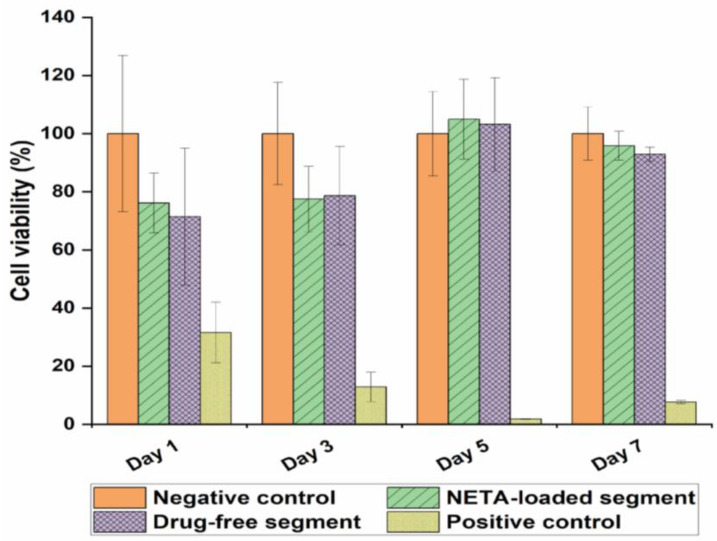
Viability of NIH/3T3 cells by MTT assay in the presence of the optimal drug-free and NETA-loaded formulations.

**Figure 12:**
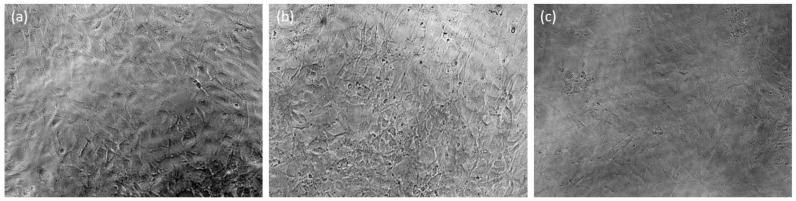
Micrographs of NIH/3T3 cells after 7 days treatment at 20× for (**a**) the negative control in cell culture media, (**b**) the NETA-loaded segment extract, and (**c**) drug-free segment extract (scale: 1 cm = 100 µm).

On Days 1, 5, and 7, both NETA-loaded and drug-free segments exhibited non-significant impact on the cellular viability, when compared to the negative control [*p* = 0.092, *p* = 0.09 (Day 1), *p* = 0.863, *p* = 0.966 (Day 5), *p* = 0.602, *p* = 0.146 (Day 7) for NETA-loaded and drug-free segment, respectively]. On Day 3, both segments exhibited significant differences compared to the negative control (*p* = 0.005, *p* = 0.009, for NETA-loaded and drug-free segments, respectively). Additionally, the results showed that there are no statistically significant differences (*p* > 0.05) between the NETA-loaded and the drug-free segment on the cellular viability throughout the 7-day study period. Adhering to regulatory guidelines, a relative cell viability exceeding 70% compared to the control group (100%) implies non-cytotoxicity of the materials [64,65]. The MTT assay results on the NIH/3T3 cell line, as illustrated in Figure 11, affirms the cytocompatibility of the prepared optimized drug-free and NETA-loaded segments. Figure 12 further represents microphotographs of NIH/3T3 on Day 7 after treatment for the negative control, NETA-loaded segment extract, and drug-free segment extract, which further demonstrates the cytocompatibility of the optimal NETA-loaded formulation.

## 4. Limitations and Future Recommendations of the Study

The undertaken study has identified a few limitations which may be considered for future research. These include the composition and volume of the simulated SUF used for drug release and weight loss assessments, which may not precisely mimic the complexity and protein content of real uterine fluid, which contains multiple proteins such as γ-globulin and hemoglobin. Additionally, the presence of proteins in the release medium could serve as a nutritional source for microorganisms, potentially leading to bio-fouling of the prepared platform and influencing the release and weight loss profiles. Furthermore, the stability of prepared NETA-loaded formulation over a prolonged period of time under different environmental conditions, including storage, was not undertaken in this study. Nevertheless, further investigations to assess the performance of the optimal NETA-loaded formulation under varying conditions, including alterations in simulated uterine fluid (SUF) compositions through the use of more biorelevant media, volume, pH values, and agitation speeds, in addition to stability studies over time could offer valuable insights into the broader applicability and robustness of the findings presented in the current study.

## 5. Conclusions

In this study, polymer-based hollow, cylindrical platforms loaded with NETA were fabricated using wet melting processing for use as a counter-estrogenic intervention in the treatment of GSM. The intricate interplay between drug loading and the proportion of matrix-forming polymers emerged as a key factor governing the kinetics of drug release and the weight loss profile in these platforms. The application of factorial experimental design stands out as an exceptional methodology, allowing for the statistical identification of experimental factor combinations and interactions critical to the attributes of the prepared platform, while the incorporation of the drug and an augmentation in the EC-to-PCL ratio significantly improved the segment strength and resistance to indentation. Moreover, the drug release mechanism from the optimized formulation revealed a sophisticated interplay between diffusion and polymer relaxation behaviors, with the fabricated platform demonstrating commendable cytocompatibility, further emphasizing its potential application as a biocompatible and implantable intrauterine device in the management of GSM. Additional research can However, be undertaken on the developed platforms including the incorporation of a sustained-release estrogenic segment for the enhanced treatment of GSM, as well as cytocompatibility studies using additional genitourinary cell lines. The developed platform can further be explored as a potential device for contraception in women of child-bearing age. These compelling findings therefore position the developed platform as a promising candidate for the sustained release of NETA within the uterine cavity to act as a counter-estrogenic intervention in the treatment of GSM, as well as potentially for other advanced biomedical applications.

## Figures and Tables

**Table 1:** Formulation compositions as generated by the 2^2^ FD.

Point Type	Formulation Code	Drug to Polymer Ratio	EC-to-PCL Ratio
Factorial	N1	5%	10%
Factorial	N2	10%	10%
Factorial	N3	5%	50%
Factorial	N4	10%	50%
Center	N5	7.5%	30%
Center	N6	7.5%	30%
Center	N7	7.5%	30%

**Table 2:** Diameter and drug content in the fabricated NETA-loaded segments (n = 3).

**Formulation**	**Diameter (mm)**	**Theoretical Loading %**	**Drug Content (% ± SD)**	**Incorporation Efficiency (% ± SD)**
N1	3.84 ± 0.006	5	4.99 ± 0.07	99.81 ± 1.36
N2	3.73 ± 0.020	10	9.98 ± 0.1	99.77 ± 1.01
N3	3.79 ± 0.080	5	5.00 ± 0.12	100.01 ± 2.33
N4	4.11 ± 0.050	10	10.03 ± 0.22	100.36 ± 2.15
N5	3.99 ± 0.076	7.5	7.51 ± 0.13	100.07 ± 1.75
N6	4.03 ± 0.021	7.5	7.50 ± 0.39	99.98 ± 5.24
N7	4.02 ± 0.012	7.5	7.48 ± 0.23	99.74 ± 3.01

**Table 3:** 2^2^ FD matrix and results for NETA-loaded tubular segments (n = 3, SD).

Formulation	1-Week Drug Release (%)	2-Week Drug Release (%)	3-Week Drug Release (%)	4-Week Drug Release (%)	8-Week Weight Loss (%)
N1	40.8 ± 3.2	62.4 ± 4.6	73.2 ± 3.9	83.7 ± 1.8	3.4 ± 0.3
N2	32.4 ± 2.8	55.3 ± 4.2	69.8 ± 5.2	78.0 ± 5.3	4.5 ± 0.2
N3	23.6 ± 1.0	37.8 ± 0.8	48.1 ± 0.6	58.6 ± 0.7	1.9 ± 0.0
N4	17.3 ± 0.9	26.9 ± 1.2	33.1 ± 1.6	38.0 ± 1.6	2.4 ± 0.0
N5	29.8 ± 1.6	46.8 ± 1.9	59.0 ± 1.9	67.0 ± 1.8	2.9 ± 0.1
N6	29.6 ± 2.3	44.7 ± 2.3	55.0 ± 2.5	62.5 ± 2.4	3.0 ± 0.1
N7	29.3 ± 2.4	44.5 ± 2.0	56.9 ± 2.4	66.0 ± 2.2	3.1 ± 0.0

**Table 4:** Model statistical fit parameters.

Formulation	1-Week Drug Release (%)	2-Week Drug Release (%)	3-Week Drug Release (%)	4-Week Drug Release (%)	8-Week Weight Loss (%)
R^2^	0.9907	0.9913	0.9679	0.9706	0.9883
Adj R^2^	0.9860	0.9870	0.9519	0.9558	0.9825
Pred R^2^	0.9515	0.9587	0.8845	0.8501	0.9497
Adeq precision	41.740	41.403	19.932	22.399	36.771
C.V.%	2.97	2.87	13.28	8.64	3.31
PRESS	15.44	32.52	1.068 × 10^10^	2.928 × 10^6^	0.022

**Table 5:** Statistical assessment of fitting mathematical kinetic models to release data from the optimized NETA-loaded segment.

Model	R^2^	Adj-R^2^	RMSE	AIC	MSC
Zero-order	0.791	0.791	4.583	179.530	1.496
First-order	0.899	0.899	3.196	159.350	2.217
Higuchi	0.975	0.975	1.602	120.660	3.599
Korsmeyer-Peppas	0.990	0.989	1.037	97.284	4.433
Peppas-Sahlin	0.997	0.997	0.575	65.168	5.580

## Data Availability

Data available on request from the authors.

## Supplementary Materials

The following supporting information can be downloaded at: https://www.mdpi.com/article/10.3390/pharmaceutics16050587/s1, Figures S1–S4: Wavescans of NETA and EC/PCL solutions at 240 nm in acetonitrile:methanol solution and SUF.
