# Supplementary material for: A Novel Intrauterine Device for the Spatio-Temporal Release of Norethindrone Acetate as a Counter-Estrogenic Intervention in the Genitourinary Syndrome of Menopause
Source: Pharmaceutics. 2024 Apr 26;16(5):587. doi: 10.3390/pharmaceutics16050587 (PMC11124343; doi:10.3390/pharmaceutics16050587)
Supplement: Supplementary file 1 [file pharmaceutics-16-00587-s001.zip › pharmaceutics-2897728-supplementary.pdf]

pharmaceutics

*Supplementary Information*

# A Novel Intrauterine Device for the Spatio-Temporal Release of Norethindrone Acetate as a Counter-Estrogenic Intervention in the Genitourinary Syndrome of Menopause

**Ahmed Abdelgader, Mershen Govender, Pradeep Kumar and Yahya E. Choonara ***

Wits Advanced Drug Delivery Platform Research Unit, Department of Pharmacy and Pharmacology, School of Therapeutic Sciences, Faculty of Health Sciences, University of the Witwatersrand, Johannesburg, 7 York Road, Parktown 2193, South Africa
* Correspondence: yahya.choonara@wits.ac.za; Tel.: +27-11-717-2052

## S1. Wavescan of NETA and EC/PCL in Acetonitrile:Methanol

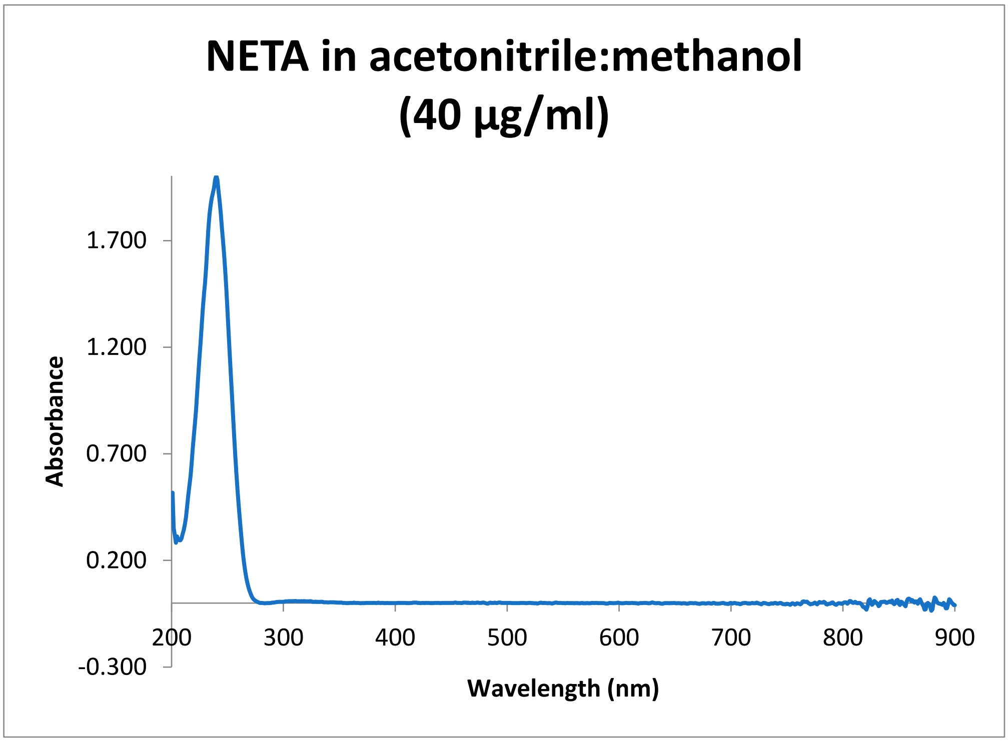


**Figure S1.** Wavescan of NETA in acetonitrile:methanol at 40 µg/ml.

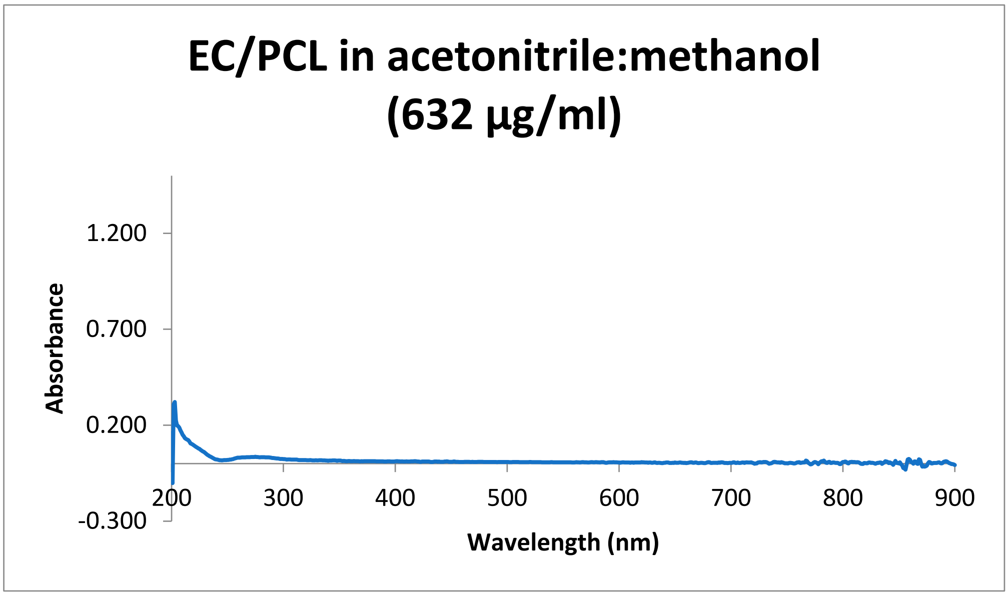


**Figure S2.** Wavescan of EC/PCL in acetonitrile:methanol at 632 µg/ml.

**S2. Wavescan of NETA and EC/PCL in SUF**

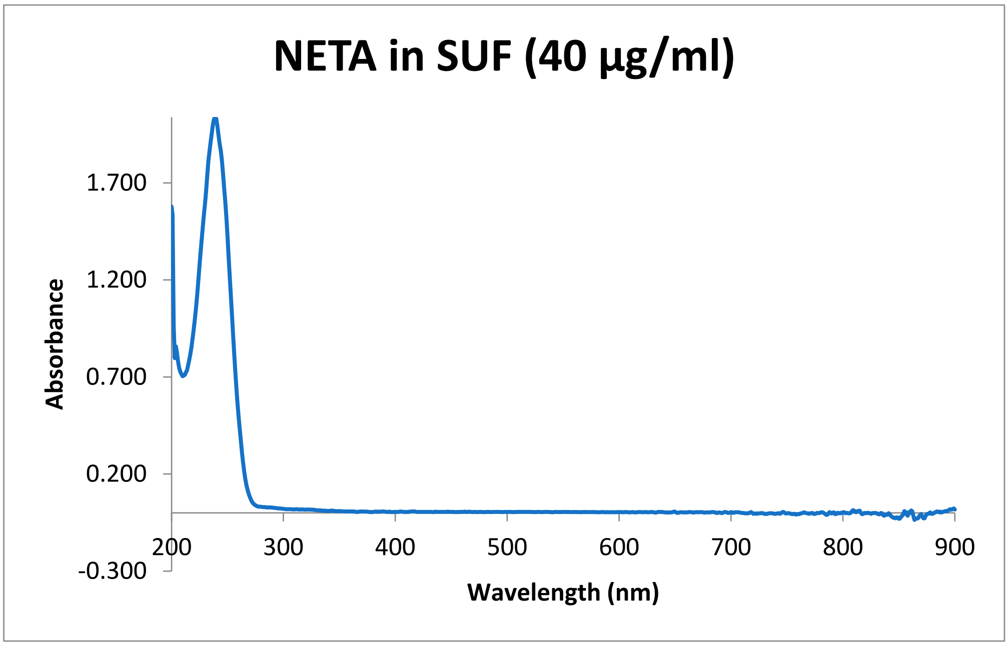


**Figure S3.** Wavescan of NETA in SUF at 40 µg/ml.

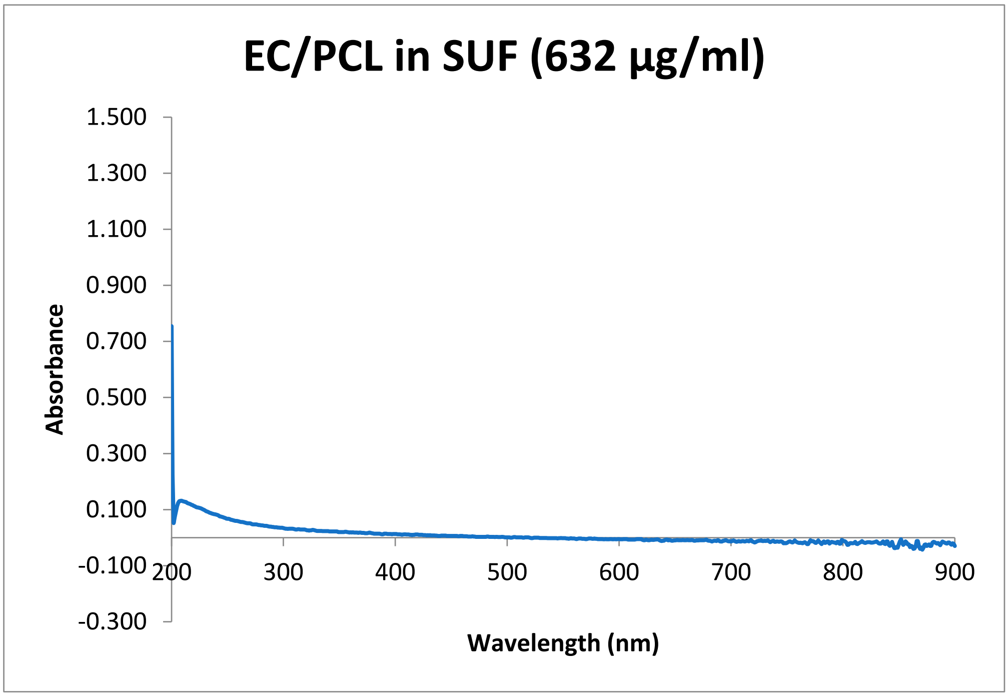


**Figure S4.** Wavescan of EC/PCL in SUF at 632 µg/ml.
